# Supplementary material for: Linear convergence of the NQZ algorithm for finding the H-spectral radius of nonnegative tensors
Source: PLoS One. 2026 Jan 23;21(1):e0338496. doi: 10.1371/journal.pone.0338496 (PMC12829971; doi:10.1371/journal.pone.0338496)
Supplement: Attachment — Submitted filename: Referee_Report.pdf [file pone.0338496.s001.pdf]

Referee Report

Manuscript Title: *Linear Convergence of the NQZ Algorithm for Finding the H-spectral Radius of Nonnegative Tensors*
Authors: Hongbin Lv, and Meixiang Chen
Journal: PLOS ONE

Summary

This study establishes the R-linear convergence of the NQZ algorithm for certain weakly irreducible nonnegative tensors using directed graphs. However, the organization and structure of the manuscript, as well as several other aspects, require attentions, as detailed below.

General Comments

(1) Page 9, Lemma 3.2:

  (a) The definition of $A^{(k)}(\theta_k)$ should be clearly provided.

  (b) In the equation preceding (2), please clarify the meaning of $\lambda_{t_1}^{k}$.

  (c) In the third equality, the notations $\bar{\pi}$ and $\tilde{i}_1$ are not defined; please explain their meaning.

  (d) On page 10, the statement "So by (3) and (4), we obtain ..." does not logically lead to equation (6); it appears that equation (6) cannot be directly obtained from (3) and (4) as claimed.

  (e) Notations need to be stated more clearly. For example, after the directed path γ, it is unclear what $a_{t_1\pi(i_2\cdots\tilde{i}_1\cdots i_m)}$ denotes. Please provide a precise definition.

(2) On page 9, Lemma 3.1: It should be carefully checked whether both $\bar{\lambda}^{(k)}$ and $\underline{\lambda}^{(k)}$ are indeed monotonically increasing.

(3) Theorem 3.1:

  (a) In the first line of the proof, it is unclear how Theorem 2.2 implies $\underline{\lambda} = \rho(\mathcal{A})$. Please clarify this reasoning.

  (b) In Algorithm 1 (NQZ Algorithm), only $\underline{\lambda}^{(k+1)}$ and $\bar{\lambda}^{(k+1)}$ are defined; the definition of $\boldsymbol{\lambda}^{(k+1)}$ should be explicitly provided.

(c) On page 12, item (i) refers to "Equation (8)", but Equation (8) appears on page 14, which seems logically inconsistent. Please revise for clarity.

(d) Please verify whether $0 < \delta < 1$ holds and provide justification.

(4) Both in Lemma 3.2 and Theorem 3.1, the exponent $\frac{(m-1)^{n-1}-1}{m-2}$ appears. However, in the matrix case ($m = 2$), the denominator becomes zero. Please clarify how this situation should be handled.

(5) Only a single, relatively simple example is presented. It is recommended to include additional examples, particularly of higher order, to better demonstrate that the results hold under more general conditions. A deeper analysis of these cases would also needed.

(6) The overall structure of the paper requires improvement. Additionally, further illustration of each theorem would enhance the reader's understanding. It is also important to explicitly state the benefits of providing an upper bound for the root convergence factor R.

(7) Some typos:

(a) On page 2, line 17, "algorithmfor" should be corrected.

(b) On page 5, Definition 2.3, item 2, the notation should not be a_{ij}.

(c) On page 7, line 10, the phrase "sequence of vertices $\gamma = \cdots$" should be revised for clarity.
