# Supplementary material for: Linear convergence of the NQZ algorithm for finding the H-spectral radius of nonnegative tensors
Source: PLoS One. 2026 Jan 23;21(1):e0338496. doi: 10.1371/journal.pone.0338496 (PMC12829971; doi:10.1371/journal.pone.0338496)
Supplement: Attachment — Submitted filename: renamed_a210c.pdf [file pone.0338496.s002.pdf]

Dear Reviewer:

Thank you for your letter and comments concerning our manuscript entitled "Linear Convergence of the NQZ Algorithm for Finding the H-spectral Radius of Nonnegative Tensors" (PONE-D-25-25102). Those comments are all valuable and very helpful for revising and improving our paper. After checking, we have modified some grammatical errors and statement expressions, please see the red font in the text. The main corrections in the revised paper and the responses to the reviewer's comments are as follows.

Responds to the reviewer's comments and suggestions:

1. Page 9, Lemma 3.2:

(1)**Comment:** The definition of $A^{(k)}(\theta_k)$ should be clearly provided.

Answer: We have checked and found that the definition of $A^{(k)}(\theta_k)$ is incorrect operation, So, we have deleted the incorrect definition in the revised paper.

(2) **Comment:** In the equation preceding (2), please clarify the meaning of $\lambda_i^{(k)}$.

Answer: The meaning of $\lambda_i^{(k)}$ is explained in detail on page 5, line 144 of the text for your further reference.

(3)**Comment:** In the third equality, the notations $\bar{\pi}$ and $\tilde{i}$ are not defined; please explain their meaning.

Answer:The notation $\bar{\pi}$ has been removed from the proof after modification, and the meaning of $\tilde{i} \in \langle n \rangle$ has been explained on page 5, line 142 of the text.

(4) **Comment:** On page 10, the statement "So by (3) and (4), we obtain ..." does not logically lead to equation (6); it appears that equation (6) cannot be directly obtained from (3) and (4) as claimed.

Answer: We have carefully checked and found there was a reference error. Now, we have corrected the logical derivation.

(5) **Comment:** Notations need to be stated more clearly. For example, after the directed path γ, it is unclear what $a_{t_1\pi(i_2,\cdots,\tilde{i}_1,\cdots,i_m)}$ denotes. Please provide a precise definition.

Answer: $a_{t_1\pi(i_2,\cdots,\tilde{i}_1,\cdots,i_m)} > 0$ is a non-zero element of A, where $\tilde{i}_0, \tilde{i}_1, \cdots, \tilde{i}_r \in \gamma$. We have added a more precise definition in the revised manuscript to clarify this notation.

2.Comment: On page 9, Lemma 3.1: It should be carefully checked whether both $\bar{\lambda}^{(k)}$ and $\underline{\lambda}^{(k)}$ are indeed monotonically increasing.

Answer: We have rechecked the monotonicity of $\bar{\lambda}^{(k)}$ and $\underline{\lambda}^{(k)}$, The proof

demonstrating that both $\overline{\lambda}^{(k)}$ and $\underline{\lambda}^{(k)}$ are monotonically increasing can be found in Theorem 2.4 of reference [3].

3. Theorem 3.1:

(1) **Comment:** In the first line of the proof, it is unclear how Theorem 2.2 implies $\underline{\lambda} = \rho(A)$. Please clarify this reasoning.

Answer:The explanation is provided on page 8, lines 193-194 of the text for your reference.

(2)**Comment:** In Algorithm 1 (NQZ Algorithm), only $\overline{\lambda}^{(k+1)}$ and $\underline{\lambda}^{(k+1)}$; the definition of $\lambda^{(k+1)}$ should be explicitly provided.

Answer:To explicitly provide the definition of $\lambda^{(k+1)}$, we have added a detailed explanation in the revised manuscript. Moreover, the relevant description is also presented on page 5, line 144 of the text for your convenient reference.

(3) **Comment:** On page 12, item (i) refers to "Equation (8)", but Equation (8) appears on page 14, which seems logically inconsistent. Please revise for clarity.

Answer: We have identified and corrected the reference error. In the revised manuscript, it is 'Equation (8)' on page 7，line178-179.

(4) **Comment:** Please verify whether $0 < \delta < 1$ holds and provide justification.

Answer: From $\underline{a} < \overline{\lambda}^{(0)}$, it can be inferred that $0 < \delta < 1$ naturally holds, as shown in line 201 on page 8 of the text.

(5) **Comment:** Both in Lemma 3.2 and Theorem 3.1, the exponent $\frac{(m-1)^{n-1}-1}{m-2}$ appears. However, in the matrix case ($m = 2$), the denominator becomes zero. Please clarify how this situation should be handled.

Answer: We have added a supplementary discussion on the case when $m = 2$ to clarify how to handle the situation where the denominator becomes zero. This supplementary content is integrated into Lemma 3.2 and Theorem 3.1 in the revised manuscript, providing a detailed explanation for this special case.

4. **Comment:** Only a single, relatively simple example is presented. It is recommended to include additional examples, particularly of higher order, to better demonstrate that the results hold under more general conditions. A deeper analysis of these cases would also needed.

Answer: We have taken your suggestion into account. In addition to the existing example, we have added a high - order example demonstrating the linear convergence of the NQZ algorithm, which is presented as Example 3.2.

5. **Comment:** The overall structure of the paper requires improvement. Additionally, further illus tration of each theorem would enhance the reader's understanding. It is also important to explicitly state the benefits of providing an upper bound for the root convergence factor *R*.

Answer: Remark 3.1 illustrates the upper bound of the root convergence factor *R*.

6. Some typos:

(1)**Comment:** On page 2, line 17, "algorithmfor" should be corrected.

Answer: We have corrected the typo "algorithmfor" on page 2, line 42 to the correct form in the revised manuscript.

(2) **Comment:** On page 5, Definition 2.3, item 2, the notation should not be a_{ij}.

Answer: We have modified the notation a_{ij} to $(M(A))_{ij}$ in Definition 2.3, item 94-96 on page 3 as suggested, and this change is reflected in the revised paper.

(3) **Comment:** On page 7, line 10, the phrase “sequence of vertices $\gamma = \cdots$” should be revised for clarity.

Answer: Modified to “sequence of vertices $\gamma : \cdots$”.To enhance clarity, we have revised the phrase “sequence of vertices $\gamma = \ldots$" on page 4, line 130 to “sequence of vertices $\gamma : \cdots$ ” in the revised manuscript.

7. We have updated the project number for research support， the National Natural Science Foundation of China in the form of a grant [61772292] and the Fujian Provincial Natural Science and Technology of China in the form of a grant [2023J01997,2024J01874] , and match ‘Funding Information’ and ‘Financial Disclosure’ sections as well.

8. The corresponding author has been updated with ORCID number 0000-0002-1654-3195.

9. The revised draft has been modified in accordance with the format specified in The PLOS ONE Style Templates, and DOIs have been added to the references.
