# Supplementary material for: Linear convergence of the NQZ algorithm for finding the H-spectral radius of nonnegative tensors
Source: PLoS One. 2026 Jan 23;21(1):e0338496. doi: 10.1371/journal.pone.0338496 (PMC12829971; doi:10.1371/journal.pone.0338496)
Supplement: Attachment — Submitted filename: Referee_Report_R1.pdf [file pone.0338496.s003.pdf]

Referee Report

Manuscript Title: *Linear Convergence of the NQZ Algorithm for Finding the H-spectral Radius of Nonnegative Tensors-R1*
Authors: Hongbin Lv, and Meixiang Chen
Journal: PLOS ONE

Summary

This study establishes the R-linear convergence of the NQZ algorithm for certain weakly irreducible nonnegative tensors using directed graphs. The authors have addressed some concerns from earlier feedback, but some issues still require attention.

General Comments

(1) Please take a close look at [3]. The sequence $\bar{\lambda}^{(k)}$ should be decreasing, as illustrated in the following figure corresponding to Theorem 2.4 of [3].

Assume that λ_0 is the unique positive eigenvalue corresponding to a nonnegative eigenvector. Then,

$$\underline{\lambda}_1 \leq \underline{\lambda}_2 \leq \cdots \leq \lambda_0 \leq \cdots \leq \bar{\lambda}_2 \leq \bar{\lambda}_1.$$

Proof. Clearly, by Theorem 1.4, for $k = 1, 2, \dots,$

$$\underline{\lambda}_k \leq \lambda_0 \leq \bar{\lambda}_k.$$

We now prove for any $k \geq 1$

$$\underline{\lambda}_k \leq \underline{\lambda}_{k+1} \quad \text{and} \quad \bar{\lambda}_{k+1} \leq \bar{\lambda}_k.$$

Figure 1: Theorem 2.4 of [3]

(2) For the two given examples, it would be better to provide convergence plots to illustrate that the algorithm exhibits linear convergence in these cases.
