# Supplementary material for: Linear convergence of the NQZ algorithm for finding the H-spectral radius of nonnegative tensors
Source: PLoS One. 2026 Jan 23;21(1):e0338496. doi: 10.1371/journal.pone.0338496 (PMC12829971; doi:10.1371/journal.pone.0338496)
Supplement: Attachment — Submitted filename: A letter to the reviewer.pdf [file pone.0338496.s004.pdf]

Dear Reviewer:

Thank you for your letter and comments concerning our manuscript entitled "Linear Convergence of the NQZ Algorithm for Finding the H-spectral Radius of Nonnegative Tensors" (PONE-D-25-25102). Those comments are all valuable and very helpful for revising and improving our paper.

After checking, Lemma 3.1 is the Theorem 2.4 in Reference [3], and it is confirmed that $\bar{\lambda}(k)$ is indeed monotonically decreasing, which has been modified in the paper. Secondly, regarding the linear convergence of the NQZ algorithm in Lemmas 3.1 and 3.2, we plotted Figure 1 for verification. In addition, we added a paragraph explaining Figure 1 at the end of Section 3.

Please review the revised version of the paper for details.Thank you.
